# X-Chromosome Inactivation and Autosomal Random Monoallelic Expression as “Faux Amis”

**DOI:** 10.3389/fcell.2021.740937

**Published:** 2021-09-23

**Authors:** Vasco M. Barreto, Nadiya Kubasova, Clara F. Alves-Pereira, Anne-Valerie Gendrel

**Affiliations:** ^1^Chronic Diseases Research Centre, CEDOC, Nova Medical School, Lisbon, Portugal; ^2^Department of Genetics, Smurfit Institute of Genetics, Trinity College Dublin, University of Dublin, Dublin, Ireland; ^3^Instituto de Medicina Molecular João Lobo Antunes, Faculdade de Medicina da Universidade de Lisboa, Lisbon, Portugal

**Keywords:** X-chromosome inactivation, random monoallelic expression, epigenetic silencing, LINE-1 elements, cellular diversity, stochasticity, dosage compensation

## Abstract

X-chromosome inactivation (XCI) and random monoallelic expression of autosomal genes (RMAE) are two paradigms of gene expression regulation where, at the single cell level, genes can be expressed from either the maternal or paternal alleles. X-chromosome inactivation takes place in female marsupial and placental mammals, while RMAE has been described in mammals and also other species. Although the outcome of both processes results in random monoallelic expression and mosaicism at the cellular level, there are many important differences. We provide here a brief sketch of the history behind the discovery of XCI and RMAE. Moreover, we review some of the distinctive features of these two phenomena, with respect to when in development they are established, their roles in dosage compensation and cellular phenotypic diversity, and the molecular mechanisms underlying their initiation and stability.

## Introduction

In diploid organisms, the two alleles of a gene are usually expressed. However, the expression levels of each allele are not necessarily equal, and allelic imbalance (AI) in transcript levels can occur due to genetic differences in the regulatory sequences or the stability of the transcripts. There are, however, special cases not explained by in *cis* differences in the sequence of the alleles. These have been lumped under the umbrella term “monoallelic expression.” In a broad sense, all genetic expression is epigenetic, but if we use a conservative definition of epigenetics to include all heritable (during mitosis or meiosis) changes in gene expression that occur without any changes in the underlying DNA sequence, then monoallelic expression becomes the poster child of epigenetics. Known cases of monoallelic expression include genomic imprinting, X-chromosome inactivation (XCI), and random monoallelic autosomal expression (RMAE). Genomic imprinting affects all cells of an organism the same way, i.e., it is always the same allele that is expressed, depending on the parent of origin (Barton et al., 1984; McGrath and Solter, 1984; Surani et al., 1984). The fate (expression or silencing) is defined during the formation of the gametes in the progenitor. Thus, despite the associated fascinating molecular mechanisms, evolutionary theory (Haig, 1993), and relevance for development and diseases (Ferguson-Smith and Bourc’his, 2018), genomic imprinting is merely a case of transgenerational gene expression that is reset each generation during the formation of the oocyte and sperm cells. XCI and RMAE differ from genomic imprinting because they give rise to mosaicism: in the same organism, some cells express the maternal allele and other cells express the paternal allele. Over the last decades, this common feature has recurrently tempted many to draw parallels between XCI and RMAE, both in reviews or opinion pieces [e.g., (Efstratiadis, 1995; Goldmit and Bergman, 2004; Chess, 2016; Gendrel et al., 2016)] and original articles [e.g., (Mostoslavsky et al., 2001; Pereira et al., 2003)]. But much like the confusion created by false cognates or “faux amis” between two languages, the parallels between two phenomena often prevent us from seeing the obvious and meaningful differences; parallels can illuminate but also deceive. Thus, here we propose to critically evaluate the relevance of the parallels drawn between XCI and RMAE, and expose their key differences at the cellular and molecular levels.

## Historical Background

XCI and RMAE were described in the same decade. X-chromosome inactivation, also named “Lyonisation,” was first proposed in 1961 by mouse geneticist Mary Lyon in a short report with no figures, where she laid the fundamental principles of XCI based solely on mouse genetics and earlier cytological evidence (Lyon, 1961). A few years later, individual B cells were shown to express only one immunoglobulin allele, both for the heavy and the kappa chains (Cebra and Goldstein, 1965; Pernis et al., 1965), two autosomal genes. Retrospectively, these three papers were seminal, but Lyon’s work immediately created a new field, whereas the RMAE of antigen receptors remained essentially a pet subject for a niche of scientists.

Mary Lyon was examining the inheritance and the phenotype of different mutations in X-linked genes affecting coat color in mice. She observed that heterozygous females had mosaic or variegated phenotypes, with patches of normal and mutant color, unlike males. This, coupled with the knowledge that female mice with only one X chromosome were viable and fertile (Welshons and Russell, 1959) and that female cells exhibit one condensed X chromosome in their nuclei (Ohno et al., 1959; Ohno and Hauschka, 1960), led her to the XCI hypothesis. The key principles underlying this hypothesis were the genetic inactivation of the X chromosome of either paternal or maternal origin, the early inactivation during embryogenesis, and the clonal inheritance of the inactive state through cell division (Lyon, 1961). Soon after, other scientists correlated the genetic observations made by Mary Lyon with experimental studies, such as the presence of two red blood cell populations or protein variants associated with mutations in the *G6pd* X-linked gene in female cells (Beutler et al., 1962; Davidson et al., 1963). In 1962, Mary Lyon published a much longer report focusing on human X-linked syndromes, providing evidence that XCI is present in other mammals, such as humans, and is the basis for dosage compensation between the sex chromosomes (Lyon, 1962). XCI is still often referred nowadays to as the “Lyon hypothesis,” although it should be considered as a fully established law (Gendrel and Heard, 2011).

The finding that the immunoglobulin chains are expressed monoallelically at the cellular level predates the discovery of the mechanism of V(D)J recombination that sets apart the antigen receptor genes (Hozumi and Tonegawa, 1976), including the immunoglobulin and T-cell receptor genes. Over the years, it was found that monoallelic expression – more commonly described in this literature as “allelic exclusion” – is a feature of most antigen receptor genes only partly explained by the relatively high frequency of non-productive sequences (with frameshifts leading to premature stop codons) generated by V(D)J recombination and that the percentage of cells with monoallelic expression varies considerably depending on the antigen receptor gene [reviewed in Vettermann and Schlissel (2010)].

The collection of genes under allelic expression expanded beyond the antigen receptor genes only in the 1990s. The olfactory receptor (OR) genes form the largest gene family in mammals; in the mouse, there are 1,296 OR genes (Zhang and Firestein, 2002). Remarkably, each neuron expresses only one gene and, taking advantage of the OR gene polymorphisms found in *Mus musculus* x *M. spretus* F1 mice, it was found that, in a given neuron, only one of the two alleles of the chosen expressed gene is transcribed (Chess et al., 1994). Soon after this finding, using allele-specific antibodies, the *Ly49* genes of natural killer cells were also shown to display a monoallelic expression pattern in mice (Held et al., 1995) and a few years later, a pheromone receptor, similar to the OR genes, was shown to display RMAE in the neurons of the accessory olfactory system (Rodriguez et al., 1999).

The end of the 1990s would mark the beginning of a short controversy on the expression patterns of the interleukins (ILs). In T cells, *IL-2* was reported to be monoallelically expressed in T cells (Hollander et al., 1998), whereas for *IL-4* the expression was described as biallelic or monoallelic, depending on the clone (Bix and Locksley, 1998) or the strength of the signal delivered through the TCR (Rivière et al., 1998). Four subsequent studies on *IL-2* reached different conclusions: whereas *IL-2* was found to be biallelically expressed in human T cell clones (Chiodetti et al., 2000; Bayley et al., 2003), in mice heterozygous for an *IL-2-GFP* transgene (Naramura et al., 1998) and in single-cell RT-PCR experiments (Rhoades et al., 2000), most cells expressed both *IL-2* alleles but there were also single expressors. Overall, the data for *IL-2* seem consistent with the data for *IL-4*: under optimal and continuous stimulation, cells will tend to express both alleles, but at suboptimal levels of expression, there may be cells expressing only one of the alleles.

The list of genes under monoallelic expression grew slowly until the mid-2000s based on additional reports focused on single genes [e.g., (Endo et al., 1995); Table 1], but in 2007, the application of genome-wide transcriptomics to a collection of human lymphoblast clonal cell lines revealed that over 5% of expressed genes display patterns of random monoallelic expression across the collection of clones (Gimelbrant et al., 2007). Over the subsequent years, several independent reports on clonal cell lines confirmed that the number of genes under random monoallelic expression was higher than previously thought (Table 2).

**TABLE 1:** List of autosomal genes under random monoallelic expression reported in studies focused on single genes.

Gene	Cell type/tissue	Species	*In vitro*/ *in vivo*	Year	References
Immunoglobulin receptor genes	B and T lymphocytes	rabbit mouse	*in vivo*	1965 1976 1985	Cebra and Goldstein, 1965; Pernis et al., 1965; Hozumi and Tonegawa, 1976; Goverman et al., 1985
Olfactory receptor (OR) genes	sensory neurons	mouse	*in vivo*	1994	Chess et al., 1994
*HUMARA* (human androgen receptor) gene	colonic crypts	human	*in vivo*	1995	Endo et al., 1995
*Ly49* receptor genes	natural killer cells	mouse	*in vivo*	1995	Held et al., 1995
Interleukin genes (*IL2*, *IL4*, *IL5*, *IL10*, *IL13*)	T cells	mouse	*in vitro*	1998, 2000, 2006	Bix and Locksley, 1998; Hollander et al., 1998; Kelly and Locksley, 2000; Calado et al., 2006
*Pax5*	early progenitors and mature B cells	mouse	*in vitro*	1999	Nutt et al., 1999
*VRi2*	sensory neurons of the vomeronasal system	mouse	*in vivo*	1999	Rodriguez et al., 1999
*Nubp2, Igfals*, and *Jsap1*	bone marrow stromal cells and hepatocytes	mouse	*in vitro*	2001	Sano et al., 2001
Variable lymphocyte receptors (VLRs) genes	lymphocytes	lamprey	*in vivo*	2004	Pancer et al., 2004
Protocadherin genes	Purkinje cells	mouse human	*in vitro*/ *in vivo*	2002 2005 2006	Tasic et al., 2002; Wang et al., 2002; Esumi et al., 2005; Kaneko et al., 2006
*Tlr4*	B cells	mouse	*in vitro*	2003	Pereira et al., 2003
*KIR* genes	natural killer cells	human	*in vitro*	2003	Chan et al., 2003
*Cd4*	CD4 + lymphocytes	mouse	*in vitro*	2004	Capparelli et al., 2004
*p120 catenin*	pre-B clonal cell lines	mouse	*in vitro*	2005	Gimelbrant et al., 2005
	lymphoblastoid lines	human			
*Gfap* (*glial fibrillary acidic protein*)	cortical astrocytes	mouse	*in vitro*	2008	Takizawa et al., 2008
rDNA loci	lymphoblasts	human	*in vitro*	2009	Schlesinger et al., 2009
*Krt12*	limbal stem cells	mouse	*in vivo*	2010	Hayashi et al., 2010
*IGF2BP1*	B cells	human	*in vitro*	2011	Thomas et al., 2011
*ASAR6*	P175 cell line (derived from HTD114 fibrosarcoma cell line)	human	*in vitro*	2011	Stoffregen et al., 2011
*Cubilin*	renal proximal tubules and small intestine	mouse	*in vivo*	2013	Aseem et al., 2013
*ASAR15*	P268 cell line (derived from HTD114 fibrosarcoma cell line)	human	*in vitro*	2015	Donley et al., 2015
*Gata3*	hematopoietic stem cells and early T-cell progenitors	mouse	*in vitro*/ *in vivo*	2015	Ku et al., 2015
*FOXP2*	B lymphoblastoid cell lines and clonal T-cell lines	human	*in vitro*/ *in vivo*	2015	Adegbola et al., 2015
*Bcl11b*	T cells	mouse	*in vitro*/ *in vivo*	2018	Ng et al., 2018

**TABLE 2:** A summary of reports based on genome-wide transcriptomics analysis in different cell types.

Cell type	Experimental assay	Species	Genotypes	% of RMAE	Number of clones analyzed	References
Lymphoblastoid cells (*in vitro*)	SNP-sensitive microarrays	Human	NA	5-10	12	Gimelbrant et al., 2007
		Mouse	129S X CAST; Balb/c X C57BL/6J	15.6	11	Zwemer et al., 2012
Fibroblasts (*in vitro*)	SNP-sensitive microarrays	Mouse	129S X CAST	2.1	2	Zwemer et al., 2012
	RNA-seq	Mouse	CAST X 129S	0.52-1.9	6	Pinter et al., 2015
Neural stem cells (*in vitro*)	SNP-sensitive microarrays	Human	NA	1.4-2.0	9	Jeffries et al., 2012
	RNA-seq	Mouse	C57BL/6 X JF1	2.4	4	Li et al., 2012
	SNP-sensitive microarrays	Human	NA	0.63	3	Jeffries et al., 2016
	RNA-seq	Mouse	C57BL/6 X JF1	4.6	4	Branciamore et al., 2018
Neural progenitor cells from embryonic stem cells (*in vitro*)	RNA-seq	Mouse	C57BL/6 X CAST	3.0	6	Eckersley-Maslin et al., 2014
			129S X CAST	2.5	8	Gendrel et al., 2014
Embryonic stem cells (*in vitro*)	RNA-seq	Mouse	C57BL/6 X CAST	0.5	6	Eckersley-Maslin et al., 2014
iPSC (*in vitro*)	SNP-sensitive microarrays	Human	NA	0.88	2	Jeffries et al., 2016
Neural stem cells from iPSC (*in vitro*)	SNP-sensitive microarrays	Human	NA	0.65-0.84	2	Jeffries et al., 2016
Astrocyte-like cells (*in vitro*)	RNA-seq	Mouse	C57BL/6 X JF1	6.4	4	Branciamore et al., 2018

Technological progress allowing transcriptomics at the single-cell level revealed that stochastic bursts of transcription occurring independently at the allelic level may lead to the presence of RNA from only one of the alleles at a given time (Deng et al., 2014). We will not cover these cases because RMAE due to transcriptional bursting is not expected to be stable over time and is not observed at the clonal level.

## Homogeneous Versus Heterogeneous Phenomena

**FIGURE 1:**
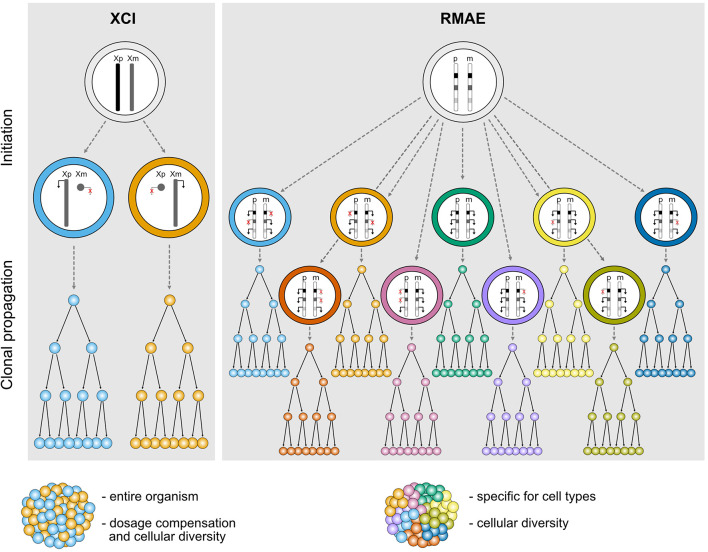
Schematic representation illustrating the features of different types of random monoallelic expression: X-chromosome inactivation (XCI) and random monoallelic autosomal expression (RMAE). Xp, X chromosome of paternal origin, Xm, X chromosome of maternal origin; p, paternal autosome, m, maternal autosome.

XCI is usually perceived as a homogeneous process. It affects an entire chromosome leading to silencing of nearly all genes, with a few notable exceptions, and therefore sets the basis for a robust monoallelic expression of these genes. The inactivation is established in all cells in a random manner early during embryogenesis and is then stably inherited during mitotic cell divisions throughout life; all cells therefore carry an inactive X (Xi) and active X (Xa) chromosome, and females are mosaic individuals with cell populations expressing genes from either the maternal or the paternal X chromosome (Figure 1). Most genes that are subject to XCI stay stably repressed during development and adulthood, and rarely become biallelically expressed, except under specific circumstances discussed below (Galupa and Heard, 2018).

In clear contrast with XCI, which has defined physical boundaries (the X chromosome), a precise timing during development, a phylogenetic association with female marsupial and placental mammals, and a master player (the *Xist* long non-coding RNA), genes under RMAE are scattered throughout autosomal chromosomes, are expressed at different times and in different tissues, can be biallelically expressed and are found in animals other than mammals, including jawless vertebrates (Pancer et al., 2004), trypanosomes (Borst, 2002), and perhaps even in diatom algae (Hoguin et al., 2021) (Figure 1). Additionally, there is no evidence that they are regulated by a single factor and no clear molecular signature that could suggest the existence of a common mechanism regulating RMAE has emerged. Although the first examples of genes under RMAE were of cell surface receptors, which remain an over-represented class, this group is diverse in terms of function.

## Timing

XCI is initiated during early embryogenesis in mammals. In mice, random XCI starts around the time of implantation (E5.5) and is complete by E6.5 in all cells in embryonic tissues (Mak et al., 2004). In humans, the timing of XCI has been complicated to address owing to obvious difficulties to access relevant material. Nevertheless, studies showed that random XCI is initiated around the implantation stage and, compared to mice, appears to be a much more gradual process during the first four weeks of embryonic development (Tang et al., 2015; Zhou et al., 2019). This process is absolutely essential, as failure to induce XCI results in early embryonic lethality at around day 10 of development for mouse embryos (Takagi and Abe, 1990).

Whereas XCI is established early in development, RMAE can occur early or late, depending on when the gene is first expressed. For instance, in the case of OR genes, the critical events leading to RMAE, namely the stochastic expression of a single OR allele from a pool of OR genes silenced with heterochromatic marks, occurs in the maturing olfactory sensory neurons of the mouse olfactory epithelium (Magklara et al., 2011). However, for most genes under RMAE, it is unknown if the choice of which allele to express is pre-determined in progenitor cells long before the gene becomes expressed. In 2001, that possibility was suggested for the antigen receptor genes based on the finding that these genes replicate asynchronously (Mostoslavsky et al., 2001). Asynchronous replication is a feature of monoallelically expressed genes because, typically, transcribed genes (or alleles) undergo replication before silenced genes (or alleles). The authors drew parallels between XCI and the pattern of asynchronous replication in autosomal genes in their study. In both processes, the epigenetic marks are erased in the morula, re-established around the time of implantation randomly and then clonally maintained. However, in subsequent publications from the same group and others, these parallels fell apart (Farago et al., 2012; Alves-Pereira et al., 2014). Notably, in mice reconstituted with a single hematopoietic stem cell, it was shown that the immunoglobulin heavy chain alleles rearrange independently, i.e., without any evidence for an epigenetic mark (Alves-Pereira et al., 2014). Whether such mark is eventually established later in development and before V(D)J rearrangement is an open question. In the kappa light chain, this pre-determination has been proposed (Farago et al., 2012), but for the heavy chain no evidence was found (Alves-Pereira et al., 2014). In any case, the advantage of such clonal pre-determination long before the genes are expressed is not obvious.

## Role

The main purpose of XCI is to enable dosage compensation of X-linked genes products to correct for the imbalance between XX females and XY males in mammals (Graves, 2016). The lethality resulting from failure of XCI is a consequence of the absence of dosage compensation. However, it remains unclear whether dosage compensation is critical for all X-linked genes or only a fraction of them. It is also not known whether compensation of dosage-sensitive genes is necessary in all tissues and all developmental stages. Transcriptome analysis of pre-implantation embryos and differentiating embryonic stem cells indicate that absence of XCI leads to failure to exit the pluripotent state, aberrant expression of extra-embryonic factors, and inappropriate expression of developmental genes, which leads to compromised development and differentiation, hence early lethality (Schulz et al., 2014; Chen et al., 2016; Borensztein et al., 2017).

Besides dosage compensation, XCI is also able to generate a significant level of biological diversity both within and between female individuals (Figure 1). This was exemplified by a study that built topographic maps of XCI mosaicism at single cell resolution, using female mice carrying X-linked fluorescent reporters on each X chromosome (Wu et al., 2014). The authors observed that some organs or tissues are particularly prone to deviations from the expected 50:50 inactivation ratio. In particular, the brain stands out as one organ where the diversity conferred by XCI could have an important functional impact for the stimulus-response amplitude of neuronal networks, especially when considering heterozygosity for an X-linked gene expressed in the brain (Wu et al., 2014). Given the level of genetic variation on the human genome including the X chromosome, XCI could generate a remarkable level of intra- and inter-individual differences in the human central nervous system.

RMAE is thought to have evolved exclusively to increase the biological (or phenotypic) diversity at the cellular level. Assuming polymorphisms within a gene, heterozygous cells with a biallelic pattern of expression will be phenotypically identical, whereas partial RMAE will produce three types of cells within the organism: single paternal allele-expressing cells, single maternal-expressing cells, and biallelic expressing cells (Figure 1). The most exuberant cases of phenotypic diversity are found in the OR and antigen receptor genes. In the former, RMAE is coupled with the selection of a single gene from the largest gene family for expression at the single-cell level, leading to the generation of hundreds or thousands of different sensory olfactory neurons, depending on the species (Niimura et al., 2014). In the latter, thousands of allelic forms are generated during the organism’s life by V(D)J recombination that will be monoallelically expressed to ensure the single cell-single receptor rule, thus facilitating the processes of negative and positive selection that shape the immune repertoires. The potential phenotypic diversity for the average gene served by only two alleles is much lower than that of OR and antigen receptor genes, but for phenotypes determined by multiple genes under RMAE there is a considerable combinatory potential (3^n^ phenotypes, where n is the number of relevant polymorphic genes under RMAE). However, outside of OR and antigen receptor genes, the importance of RMAE-driven phenotypic diversity remains to be demonstrated, and it is a complicated problem to tackle experimentally. As explained below, particular *cis*-regulatory sequences play a role in RMAE. Thus, a feasible approach would be to replace these sequences with regular promoters, but even in this case the interpretation of the data would not be clear-cut because the expression of multiple receptors at the surface of the cell would decrease the density of any particular receptor compared to its density in a cell with RMAE. Since the manipulation of master epigenetic regulators is unlikely to be sufficiently specific, a proof of principle will probably be obtained using CRISPR/Cas tools that allow epigenetic manipulations at the allele-specific level. An alternative approach would be to generate aggregation chimeras of cells expressing different receptors or other relevant proteins for the quantitative response to be tested. In the absence of such data, other hypotheses can be raised, such as a role for RMAE in dosage compensation (Gendrel et al., 2016). However, the finding that genes under RMAE have increased genetic diversity (polymorphisms) in humans compared to biallelically expressed genes remains a powerful indication that RMAE evolved to increase phenotypic diversity at the cellular level (Savova et al., 2016).

## Molecular Mechanisms

### Stochasticity

One has to recognize that stochasticity is the key feature common to XCI and RMAE: how come identical or quasi-identical sequences (the X chromosomes or autosomal alleles) sharing the same nuclear environment undergo completely opposite fates (expression or silencing)? This stochastic component is at the core of the appeal these phenomena have to biologists, but there is a critical difference between XCI and RMAE worth mentioning. It has been proposed that each individual X chromosome has an independent probability to be inactivated that is directly proportional to the X: ploidy ratio. Selection in favor of cells keeping one active X chromosome per diploid genome eliminates cells with two inactive X or two active X chromosomes (Monkhorst et al., 2008; Sousa et al., 2018). Thus, XCI involves the inactivation of one X chromosome and counterselection at the cellular level. In contrast, the stochastic component in OR genes, antigen receptor genes, and possibly other autosomal genes under RMAE involves the activation of alleles in a default state of silencing, and cell counterselection is not thought to play a major role in shaping the pattern of monoallelic expression; in fact, B lymphocytes genetically engineered to express two different immunoglobulin heavy chains at the surface were shown to be fit and able to generate a normal B cell compartment (Sonoda et al., 1997).

### Feedback

A key aspect of RMAE in antigen receptor genes is the feedback mechanism that prevents the recombination of the second allele once the protein encoded by the first allele to rearrange productively is expressed at the surface. When the exon encoding the transmembrane domain of the immunoglobulin chain is disrupted, the cell is no longer able to trigger this feedback mechanism and the second allele is given the chance to recombine (Kitamura and Rajewsky, 1992). A similar mechanism has been described for the beta chain of the TCR gene (Aifantis et al., 1997) and the OR genes (Serizawa et al., 2003). The overall picture, then, is the coupling of a stochastic process of gene activation that is sufficiently slow for negative feedback mechanisms to act, preventing further rearrangements (antigen receptors) or gene activation (OR genes). Because the feedback mechanism implies a time-window during which the two alleles can be activated, it also explains the generation of biallelic cells; a slow feedback mechanism will produce many biallelic cells, whereas biallelic cells are rare when the time-window is narrow. However, it is not clear whether such feedbacks are involved for other genes under RMAE and additional mechanisms have been described, which we discuss below.

### Epigenetics

The process of XCI can be divided in two distinct stages: initiation and maintenance. During the initiation phase, XCI is dependent on the expression of the long non-coding RNA *Xist*, which induces transcriptional silencing in *cis* and ultimately coats the entire inactive X chromosome (Loda and Heard, 2019). However, *Xist* is no longer essential for the maintenance of XCI, as deletion of *Xist* in somatic cells in culture does not lead to Xi reactivation (Brown and Willard, 1994). Following *Xist* accumulation on the Xi, one of the first observable events is the formation of a 3D silent nuclear compartment excluding RNA polymerase II and transcription factors, likely to be important for *Xist* spreading and the initiation of gene silencing (Chaumeil et al., 2006; Clemson et al., 2006; Pandya-Jones et al., 2020). *Xist* interacts with several RNA-binding proteins, in particular SPEN, which acts as a bridge between *Xist* RNA and repressor complexes that mediates the removal of histone modifications associated with active genes (H3K27ac, H3K9ac, H4ac), a crucial early step for the initiation of gene silencing (Żylicz et al., 2019; Dossin et al., 2020). Following this, a number of chromosome-wide chromatin changes occur on the Xi to lock in the silenced state, such as deposition of repressive histone modifications (H2AK119Ub and H3K27me3) mediated by the Polycomb repressive complexes 1 and 2 [reviewed in Boeren and Gribnau (2021)]. The late or maintenance phase is characterized by a switch to late replication timing, incorporation of the histone variant macroH2A and DNA methylation of X-linked gene promoter regions by the DNA methyltransferase Dnmt3b [reviewed in Strehle and Guttman (2020)]. These changes ensure the stable and heritable silencing of the majority of genes on the Xi, over hundreds of cell divisions.

By definition, epigenetic modifications, namely histone modifications and DNA methylation, and non-coding RNAs, have been shown to be associated with genes under RMAE (Gendrel et al., 2016). However, unlike XCI, there is no master regulator, and several scenarios have been reported, such the initial repression of both alleles followed by activation (e.g., OR genes and murine *Ly49* genes) or the initial activation of both alleles followed by the inactivation of one allele (e.g., human *KIR* genes).

### Long Interspersed Nuclear Element-1

One puzzling question in XCI has been the nature of the X-linked *cis*-acting elements important for the binding and spreading of *Xist* along the X chromosome, prior to gene silencing. Because of the higher density of long interspersed nuclear element-1 (LINE-1) retrotransposons in the X chromosome compared to autosomes (Boyle et al., 1990; Ross et al., 2005), with her so-called “repeat hypothesis,” Mary Lyon postulated that these sequences could act as booster elements for the spreading of the inactive signal along the chromosome and efficient silencing (Lyon, 1998). However, we now know that *Xist* does not bind directly LINE-1 sequences nor associate with LINE-1-enriched regions. *Xist* rather exploits the 3D conformation of the X chromosome to spread first to sites that are spatially proximal to the *Xist* gene at the onset of XCI and is then found enriched over gene-dense regions that are depleted of LINE-1 sequences (Engreitz et al., 2013; Simon et al., 2013). Yet, studies of *Xist* spreading on autosomal chromatin in X:autosome translocations (Sharp et al., 2002; Popova et al., 2006) or using *Xist* transgenes on autosomes (Chow et al., 2010; Tang et al., 2010; Loda et al., 2017) all show a good correlation between LINE-1 density, efficiency of spreading, and gene silencing. These observations suggest that LINE-1 elements may contribute to the process of XCI, either by facilitating gene silencing locally in some regions, reinforcing the long-term maintenance of XCI and/or influencing heterochromatin reorganization. This, however, remains to be formally tested using, for example, functional approaches to perturb LINE-1 expression or enrichment on the X chromosome.

Whether genes under RMAE have a DNA sequence signature remains unclear. It has been proposed that these genes are surrounded by an increased density of LINE-1 elements, which are evolutionarily more recent and less truncated than the LINE-1 elements around biallelically expressed genes (Allen et al., 2003). How exactly LINE-1 elements could contribute to RMAE is not known, but their association to RMAE would be one of the few potential parallels with XCI. However, the overlap between predicted RMAE genes based on the presence of LINE-1 elements and the collection of genes under RMAE generated by a genome-wide approach is not statistically significant (Allen et al., 2003; Gimelbrant et al., 2007).

It has been known for decades that the X chromosome and autosomal genes under RMAE replicate asynchronously (Taylor, 1960; Chess et al., 1994) and that this mitotically stable pattern is established early in development. Asynchronous replication was even found to be a property of autosomal chromosomes (Singh et al., 2003), reinforcing the parallel with the X chromosome. Whether the asynchronous replication of autosomes is absolutely stable is not clear, and it has been found that RMAE is not coordinated at the chromosome level, i.e., the alleles from different genes under RMAE on the same chromosome can be active or silent (Gimelbrant et al., 2007). However, an autosomal gene named asynchronous replication and autosomal RNA on chromosome 6 (*ASAR6*) was shown to encode a non-coding RNA under RMAE, which when expressed leads to the silencing of nearby alleles and remains associated with the chromosome from which it is expressed. Moreover, the disruption of this locus results in delayed replication timing and reactivation of previously silent alleles of nearby genes (Stoffregen et al., 2011; Donley et al., 2013). There is an obvious parallel with *XIST*, which is also monoallelically expressed, silences most of the genes on the X chromosome in *cis* and, when deleted, also alters replication timing (Diaz-Perez et al., 2005). Interestingly, a LINE-1 retrotransposon located within *ASAR6*, in antisense orientation, was then found to control the replication timing (Platt et al., 2018). This constitutes one of the most solid evidence that LINE-1 elements could be involved in the spreading of inactivation also on autosomal chromosomes. Another locus, *ASAR15*, displays features similar with *ASAR6* (Donley et al., 2015). However, it is not known how frequent this type of regional silencing occurs on autosomes, and, unlike *XIST*, the *ASAR6* RNA does not seem to coat the entire chromosome 6 and is not expressed in adult tissues (Stoffregen et al., 2011). A thorough and granular reappreciation of the impact of LINE-1 elements in RMAE would be welcomed.

### Bivalent Promoters

**FIGURE 2:**
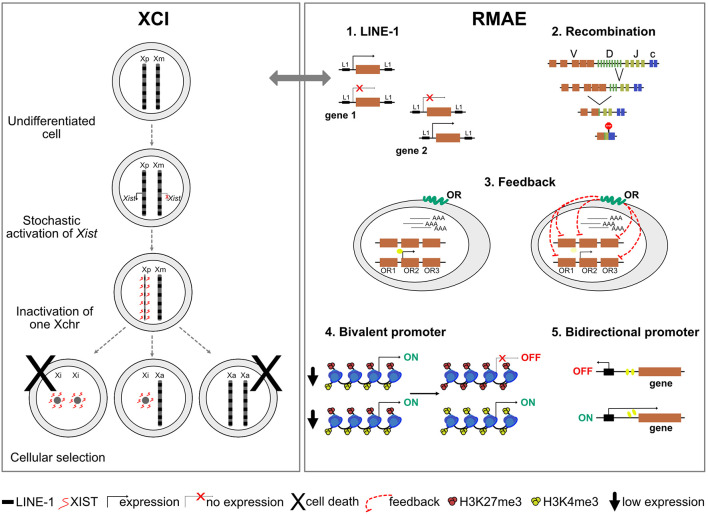
A schematic representation of mechanisms responsible for X-chromosome inactivation (XCI) and mechanisms possibly responsible for random monoallelic autosomal expression (RMAE). The gray arrow represents a potential parallel between XCI and RMAE associated with LINE-1 (L1) elements.

Several histone modifications influence gene expression, including H3K4me3 and H3K27me3, which are associated with gene activation and repression, respectively. Although active and repressive histone marks are typically imagined as being mutually exclusive, in 2006 two groups reported the existence of regulatory regions – named bivalent domains – that have both (Azuara et al., 2006; Bernstein et al., 2006). Genes with bivalent promoters in embryonic stem cells are expressed at low levels but thought to be poised for rapid activation upon differentiation cues. Interestingly, about 80% of the genes under RMAE in differentiated cells, identified by transcriptomics or the presence of activation and repression histone marks on different alleles, were found to have bivalent promoters in precursor cells (Nag et al., 2013) (Figure 2). Thus, the rapid and timely activation ensured by bivalent promoters seems to increase the probability of RMAE, as if the alleles resolve their status stochastically, leading to cells that activate only the paternal or maternal allele and cells that activate both (Nag et al., 2013).

### Bidirectional Promoters and Other Switches

Two cases of RMAE dissected in considerable detail are the murine *Ly49* receptor genes of natural killer cells and the human *KIR* genes [reviewed in Anderson (2014)]. Both rely on *cis* probabilistic bidirectional promoter switches that produce sense and antisense transcripts associated with expression and silencing, respectively (Figure 2). In the case of the murine *Ly49* receptor genes, the default condition is silencing. Transcription starts if the sense non-coding transcripts of a distal bidirectional promoter (Pro1) activate a downstream promoter (Pro2); the antisense transcripts of the bidirectional promoter do not lead to gene activation (Saleh et al., 2002, 2004). In the case of the *KIR* genes, the default condition is activation and the role of the stochastic switch, located close to the ATG start codon, is to produce a sense transcript that correlates with the maintenance of the activation state or an antisense piRNA that silences the allele. The murine *Ly49* and the human *KIR* genes illustrate how a probabilistic bidirectional promoter can create a mitotically stable asymmetry between two alleles. Whether these are two exceptional cases or examples of a frequent solution to generate RMAE has not been addressed. Notably, divergently transcribed gene pairs represent more than 10% of the human genes (Trinklein et al., 2004; Yang et al., 2007), and thorough analyses of tissue-specific sense and antisense transcripts from the same locus [e.g., (Hu et al., 2014)] could reveal additional candidate genes to be under RMAE due to bidirectional promoter or other complex arrangements of regulatory sequences leading to genetic switches.

## Stability

Once established, XCI is believed to be extremely stable and irreversible. Genes that are subject to XCI rarely show reactivation and biallelic expression, as silencing is maintained through multiple layers of epigenetic control. However, there are some exceptions and some genes can be expressed from both the Xa and the Xi. This is the case for genes that have a Y-linked homolog, including genes from the pseudoautosomal regions (PAR1 and PAR2, short regions of homology between the X and Y chromosomes, which undergo recombination during meiosis), for which there is no requirement for dosage compensation. Several genes not located in the PAR regions have also retained a functional Y paralog and would thus appear not to require dosage compensation. However, other genes do not have a Y-linked copy yet still have the ability to escape XCI (Berletch et al., 2010). In some cases, this may be due to a highly controlled process permitting escape where the gene product is required in increased dose, while in other cases, it may be due to leaky or inefficient XCI.

Escape from X inactivation is rather limited in the mouse, with around 3% of genes displaying such behavior in somatic cells (Yang et al., 2010; Pinter et al., 2012). In humans, the situation is different, as 15% of genes (excluding the PAR) have been reported to escape XCI (Carrel and Willard, 2005). Intriguingly, an additional 10% of X-linked genes appear to show heterogeneous inactivation and escape, varying between lineages and from one individual to another (Carrel and Willard, 2005). Such candidates seem to display accessible promoter regions on the Xi (Kucera et al., 2011), suggesting that they may be poised for expression in some cell lineages and that the Xi allele becomes active under specific circumstances. In the mouse, lineage-specific escape has also been found, for example in the case of the *Atrx* gene, which is fully inactivated in embryonic tissues but escapes inactivation in specific subsets of extraembryonic cells (Garrick et al., 2006). The Atrx protein is actually enriched on the Xi in extraembryonic tissues (Baumann and De La Fuente, 2009; Sarma et al., 2014), suggesting that its escape from XCI might occur in a regulated manner in tissues where a higher dose of the protein is necessary (Corbel et al., 2013).

Interestingly, some of the phenotypes observed in Turner (X0) syndrome patients are believed to be due, in part, to the reduced expression levels of escapees given the lack of the second X chromosome (Berletch et al., 2010). This indicates that expression of a double dose is essential for some X-linked genes and that escape for these genes is a highly controlled process. Escape or reactivation of genes from the Xi can also occur more sporadically, but it is currently unknown whether this is caused by inefficient XCI or associated with a controlled mechanism.

Sporadic reactivation of genes from the Xi has been observed in non-pathological contexts, in specific tissues (Gendrel et al., 2014) or lineages (Wang et al., 2016), during aging (Migeon et al., 1988; Sharp et al., 2000) and also in disease contexts (Youness et al., 2021). In normal contexts, both the brain and the lymphoid lineage appear to stand as exceptions. In the brain, the *Mecp2* gene, which is associated with Rett syndrome, was shown to display biallelic expression in a significant proportion of neural stem cells in the subventricular zone in the neonatal brain of inbred female mice (Gendrel et al., 2014). This could be indicative of a certain relaxation of epigenetic control of the Xi in these cells at least for this gene or a need for an increased dose of the protein, given that MeCP2 is a highly abundant protein in the brain (Skene et al., 2010). Moreover, *Xist* conditional deletion in adult mice leads to a global erasure of repressive histone modifications from the Xi, and more importantly, a mild loss of dosage compensation in 2-5% of neurons (Adrianse et al., 2018), highlighting again the peculiarity of the brain and neuronal lineages. Other studies have also reported partial Xi reactivation following *Xist* conditional deletion in adult tissues (Yildirim et al., 2013; Yang et al., 2016, 2020).

In the female lymphoid lineage, the maintenance of XCI is atypical and it has been hypothesized that this could predispose females to autoimmunity (Wang et al., 2016; Syrett et al., 2019). It was shown that both human and mouse naive B and T lymphocytes miss the typical *XIST*/*Xist* RNA domain within the nuclei. Instead, *Xist* shows an unusual and dispersed pattern, associated with a structure lacking some of the canonical hallmarks of heterochromatin of the Xi, such as H3K27me3, H2AK119ub1 and macroH2A. However, this state appears transient as both *Xist* and repressive histone marks are relocalized to the Xi upon B/T cell activation (Savarese et al., 2006; Wang et al., 2016; Syrett et al., 2019). This state was shown to be correlated with modest biallelic expression and increased expression of X-linked immunity-related genes (Wang et al., 2016; Souyris et al., 2018). The role of reactivation/increased expression of these genes and whether this is a cause of the atypical maintenance of XCI during lymphocyte differentiation remain unclear. However, enhanced expression of these genes could contribute to higher susceptibility of females to autoimmune disorders, such as systemic lupus erythematosus if not properly regulated (Youness et al., 2021).

Concerning RMAE, by definition all the examples of monoallelic expression discovered in clonal cell lines are mitotically stable. However, most reports have focused on cells that are also phenotypically stable, i.e., cells that during the period of the study do not go through major steps of differentiation. Thus, it remains to be addressed if RMAE is as stable as XCI, which is known to keep the status of the X chromosomes established early in development even after hundreds of cell divisions and extensive differentiation. Unfortunately, the antigen receptor and OR genes, which have been thoroughly investigated over decades, do not shed much light on this issue. The monoallelic expression pattern of antigen receptor genes is in part established by the process of V(D)J recombination and in developing lymphocytes, when recombination is active, the second allele is given the chance to recombine if the rearrangement of the first allele did not lead to the production of a receptor. In other words, the stability is not achieved before the expression of the receptor on the surface. Furthermore, the kappa immunoglobulin undergoes a process of receptor editing during which it can replace at its surface one protein by the protein encoded by the other allele (Casellas et al., 2001, 2007). It is only in mature lymphocytes that the pattern of monoallelic expression is stable, because the process of V(D)J recombination is permanently shut down and the silenced allele is epigenetically repressed and repositioned in the nucleus. With respect to OR genes, the patterns of monoallelic expression are stable, but it should be kept in mind that the cells are post-mitotic and terminally differentiated (Monahan and Lomvardas, 2015).

## Exceptions

In mammalian females, under normal physiological conditions cases of two active X chromosomes are only found in undifferentiated cells and primordial germ cells before meiosis entry. All other cells have only one active X chromosome, because the double X dosage interferes with differentiation (Schulz et al., 2014). In contrast, biallelic expression of genes under RMAE is common and ranges from rare cells, such as in the case of the immunoglobulin heavy chain (Barreto and Cumano, 2000), to biallelic populations as frequent as the monoallelic ones (Gimelbrant et al., 2007). In the case of genes under RMAE with a low frequency of biallelic expression, these exceptions could correspond to the rare cases in which the two alleles become activated within the time-window allowed, before a negative feedback is triggered. Cases with a sizable population of biallelic expression could result from a relatively high individual probability of allele activation if the fitness of the cell is not compromised by the dual expression.

## Concluding Remarks

**FIGURE 3:**
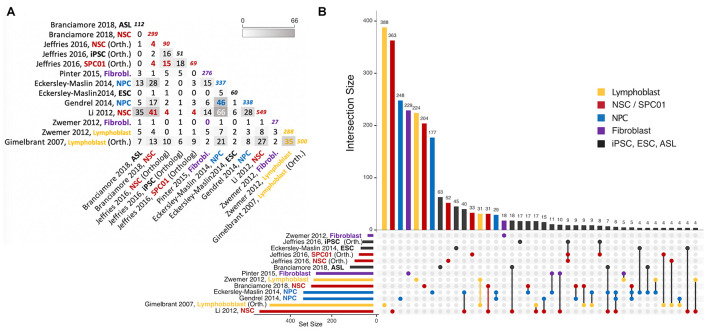
Intersections of autosomal gene collections identified as random monoallelically expressed in the genome-wide studies described in Table 2 (except Jeffries et al., 2012, which is not publicly available). **(A)** Half-matrix showing all pairwise intersections. **(B)** Upset plot (Conway et al., 2017) showing gene collection intersections of the same studies. The lower part of the panel has a horizontal bar plot showing the number of elements on each study collection, and a right section with a dot matrix. Each dot represents unique gene intersections, i.e., each gene is represented only once in the dot matrix. The upper vertical bar plot is related to the dot matrix, showing the number of unique genes in each intersection (for instance, there are 500 MAE genes in the Gimelbrant et al. (2007) dataset, but only 388 of those are uniquely present in that dataset; similarly, the Li et al. (2012) dataset shares more than 40 MAE genes with Eckerley-Maslin (2014) NPC dataset, but those 40 are uniquely shared between those two sets). Intersections of size smaller than 4 are not represented. For a complete description of the intersections and gene listing, see the Supplementary File provided with this review. ASL, Astrocyte-like cells; NSC, Neural stem cells; NPC, Neural progenitor cells; ESC, Embryonic stem cells; SPC01, Clonal Neural stem cells (before epigenetic reprogramming); iPSC, induced Pluripotent stem cells after epigenetic reprogramming of SPC01. Note that “NPC” on Jeffries et al. (2016) are derived from iPSC. Colors represent instances where a different cell/tissue type was studied more than once. To obtain intersections, gene ids were manually curated for immediate inconsistencies (e.g., gene name-to-date conversions when data was originally provided in microsoft excel format). All gene sets were then parsed with the gprofiler2 R package (Raudvere et al., 2019) for gene id consistency, using transcript ids as query whenever possible, and ENSEMBL gene ids as target (performed July 12th, 2021). Orthology conversion (from human to mouse) was performed with the same package for datasets involving human data. For Gimelbrant et al. (2007) and Zwemer et al. (2012) gene collections, MAE classes I, II and III were used to retrieve RMAE genes, and for Gendrel et al. (2014), the “NPC_random_catalog” classification was retrieved as RMAE.

X-chromosome inactivation (XCI) is a well-established specific silencing mechanism that ensures dosage compensation between the sexes in marsupial and placental mammals. At the heart of this process lies the long non-coding RNA *Xist*, which is capable of orchestrating structural changes and recruiting chromatin and repressor complexes to ensure transcriptional gene silencing at the level of an entire chromosome, early in development. XCI has clear implications in disease, as illustrated by the Turner (X0) and Klinefelter (XXY) syndromes, as well as the severe phenotypes or lethality in males and variable phenotypes in females associated with X-linked disorders (e.g., Duchenne muscular dystrophy, hemophilia, and Rett syndrome). In contrast, RMAE evolved independently in a wide range of organisms beyond mammals, mostly to increase phenotypic diversity at the cellular level. RMAE lacks a master regulator and various mechanisms can establish it at different times during cellular differentiation. The collection of target genes encompass many cell types, showing some degree of overlap (Figure 3 and Supplementary File), but have been reported to be largely tissue-specific (Gendrel et al., 2016). Finally, the extent of the bias in monoallelic expression varies widely amongst RMAE genes. In addition, there is so far no obvious link between the RMAE of autosomal genes and disease, although a number of RMAE genes are associated with autosomal dominant diseases. X-chromosome inactivation and RMAE are essentially different phenomena that share the stochastic component and perhaps the asymmetric silencing of chromosomal regions dependent on the presence of LINE-1 elements. But it is not known whether and how exactly LINE-1 elements boost XCI and if a similar process explains a considerable fraction of genes under RMAE.

## Author Contributions

VMB and A-VG wrote the manuscript. NK and CFA-P prepared the tables and figures. All authors contributed to the review content.

## Conflict of Interest

The authors declare that the research was conducted in the absence of any commercial or financial relationships that could be construed as a potential conflict of interest.

## Publisher’s Note

All claims expressed in this article are solely those of the authors and do not necessarily represent those of their affiliated organizations, or those of the publisher, the editors and the reviewers. Any product that may be evaluated in this article, or claim that may be made by its manufacturer, is not guaranteed or endorsed by the publisher.

## Abbreviations

AI: allelic imbalance
IL: interleukin
iPSC: induced pluripotent stem cell
LINE-1: long interspersed nuclear element-1
OR: olfactory receptor
PAR: pseudoautosomal region
RMAE: random monoallelic autosomal expression
SNP: single nucleotide polymorphism
TCR: T-cell receptor
Xa: active X
XCI: X-chromosome inactivation
Xi: inactive X.
